# *Operando* detection of dissolved oxygen in fluid solution using a submersible rapid scan EPR on a chip dipstick sensor

**DOI:** 10.1038/s41598-025-93591-4

**Published:** 2025-03-21

**Authors:** Joseph E. McPeak, Michele Segantini, Gianluca Marcozzi, Irene Dona, Silvio Künstner, Anh Chu, Michal Kern, Martin Poncelet, Benoit Driesschaert, Jens Anders, Klaus Lips

**Affiliations:** 1 Berlin Joint EPR Laboratory and EPR4Energy, Department Spins in Energy Conversion and Quantum Information Science (ASPIN), Helmholtz-Zentrum Berlin für Materialien und Energie GmbH, Berlin, Germany; 2 Department of Chemistry, Novo Nordisk Foundation Pulse EPR Center, University of Copenhagen, Copenhagen, Denmark; 3 Institute of Smart Sensors, Universität Stuttgart, Stuttgart, Germany; 4 Department of Pharmaceutical Sciences, School of Pharmacy, West Virginia University, Morgantown, WV USA; 5 In Vivo Multifunctional Magnetic Resonance Center, Robert C. Byrd Health Sciences Center, West Virginia University, Morgantown, WV USA; 6 Center for Integrated Quantum Science and Technology (IQST), Stuttgart and Ulm, Germany; 7 Berlin Joint EPR Laboratory, Fachbereich Physik, Freie Universität Berlin, Berlin, Germany

**Keywords:** Operando, Dipstick, Rapid scan EPR, Oximetry, EPR-on-a-chip (EPRoC), Sensor, Electrical and electronic engineering, Physical chemistry, Diagnosis

## Abstract

Electron paramagnetic resonance (EPR) is an accurate and efficient technique to probe unpaired electrons in many applications across materials science, chemistry, and biology. Dynamic processes are investigated using EPR; however, these applications are limited by the use of resonator-based spectrometers such that the entire process must be confined to the resonator. The EPR-on-a-chip (EPRoC) device circumvents this limitation by integrating the entire EPR spectrometer into a single microchip. In this approach, the coil of a voltage-controlled oscillator (VCO) is used as the microwave source and detector simultaneously, operating under a protective coating such that the device may be placed in the sample solution directly. Additionally, improvements in sensitivity via rapid scan EPR (RS-EPR/RS-EPRoC) increase the accessible applications where SNR per measurement time is the fundamental limit. The herein reported device combines a dipstick EPRoC sensor with the enhanced sensitivity of frequency-swept frequency modulated rapid scan to measure triarylmethyl (trityl, Ox071) oxygen-sensitive probes dissolved in aqueous solutions. EPR spectra of Ox071 solutions were recorded using the RS-EPRoC sensor while varying the oxygen concentration of the solution between normal atmosphere and after purging the solution with nitrogen gas. We demonstrate that EPRoC may be employed to monitor dissolved oxygen in fluid solution in an online fashion.

**Supplementary Information:**

The online version contains supplementary material available at 10.1038/s41598-025-93591-4.

## Introduction

Electron paramagnetic resonance (EPR) is a spectroscopic technique to probe the environment of unpaired electrons spins that has found use in many applications ranging from materials science, chemistry, biology, healthcare, and even medical imaging^1^. Though it is primarily employed as a tool in fundamental research, there has been growing interest over the last several decades in the adaptation of EPR methods to medical diagnostics. Assessment of local oxygen concentration is paramount in a plethora of peripheral diagnostics such as wound healing, blood oxygen content, tissue oxygenation, and in identifying cardiovascular diseases as well as for assessment of brain function^2–4^. Oxygen consumption, which is often investigated simultaneously with oxygen concentration, is also implicated in oxidative stress and plays a particularly important role in mitochondrial function^5–7^. These processes are underpinned by the production of reactive oxygen species (ROS) which cause significant cellular damage and therefore are of particular importance for the overall assessment of human health^2,5–7^. For this reason several probe molecules have been developed to interrogate these processes using EPR spectroscopy, such as solid probes like lithium phthalocyanine and soluble probes such as nitroxides and triarylmethyl (trityl) radicals^8–14^. Of these, the trityl probes have shown the most promise in clinical applications due to their increased stability in biological media^14–17^.

One of the most advantageous reporting schemes employing EPR methods is the assessment of local oxygen concentration is in the context of EPR imaging, similar to how nuclear magnetic resonance (NMR) spectroscopy has experienced widespread adoption via magnetic resonance imaging (MRI) methods routinely used in the clinic and has even been used to map oxygen concentration in tumors in both preclinical and clinical trials^16–20^. Ex-vivo methods of measuring oxygen concentration have been employed to show effects of oxygen in cell-death, antioxidant transcription, cell proliferation, and acute inflammation; and, more recently EPR imaging has been incorporated into well plates for high throughput measurements of biological samples^21,22^. While toxicity towards cells and other biological samples is a major concern, another drawback to the utilization of EPR methods in medical assessment is the requirement for a paramagnetic spin probe or reporter molecule that is sufficiently tailored to the information desired from the living organism.^1417,1916,182–45–72,5–78–1414–17^

**Fig. 1:**
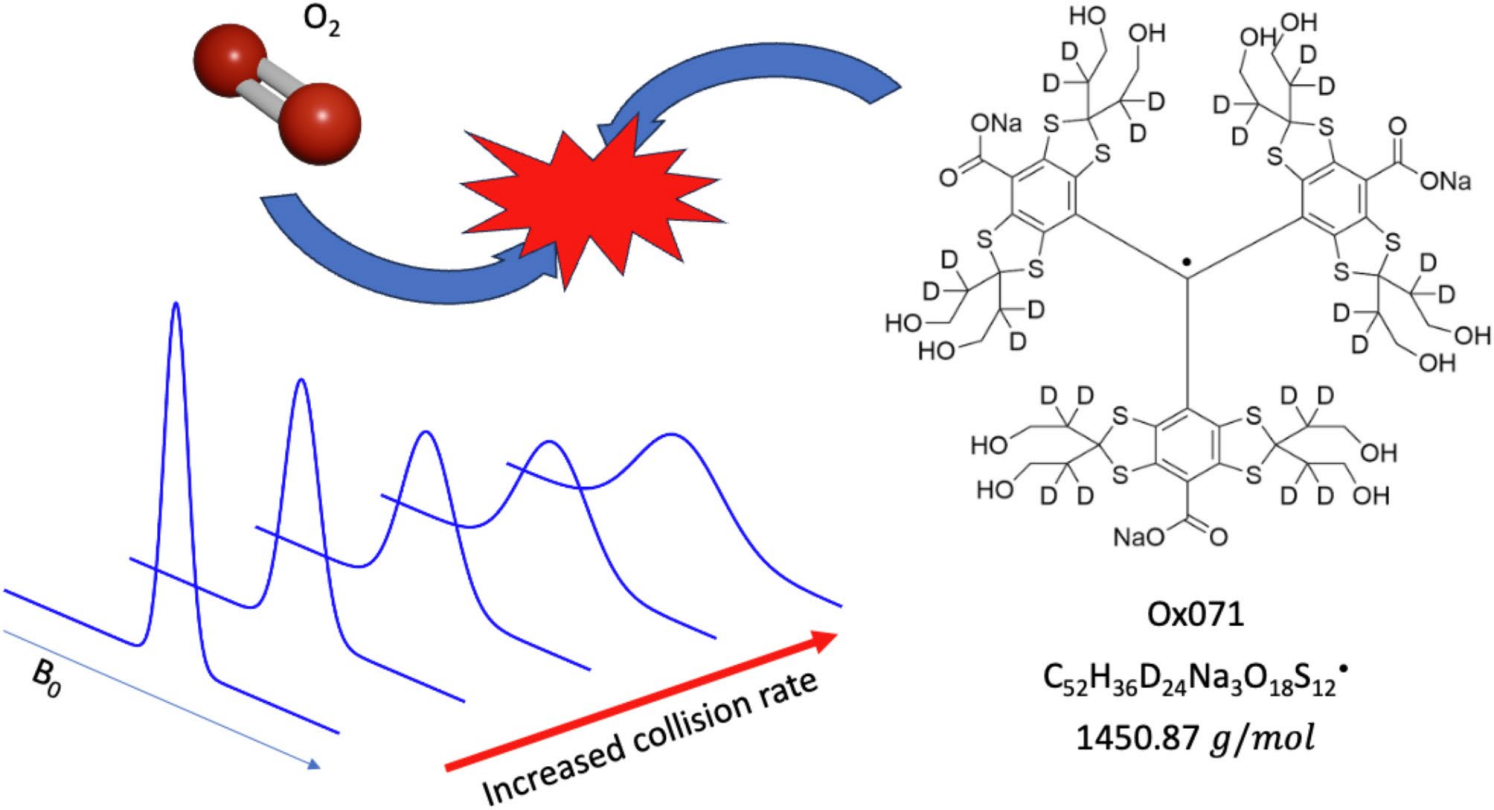
An overview of oxygen monitoring via EPR where the monitoring capabilities of the probe molecule are dependent on the collisional interactions with radical-oxygen species, detectable as a linewidth-dependent change in the relaxation rate of the trityl spin probe, Ox071.

EPR spectroscopy is well suited for the task of reporting the local concentration of oxygen via paramagnetic triarylmethyl probes (Fig. 1). While molecular oxygen possesses radical characteristics, relaxation occurs too rapidly to detect it. However, the molecular collision interaction of the carbon-centered triarylmethyl (trityl) radical probes with molecular oxygen may be accurately assessed via lineshape broadening in the trityl EPR spectra^3,15,23^. The inherently narrow lines of trityl radicals provide excellent oxygen sensitivity^24–26^. Through careful calibration, the linewidth of the trityl accurately reports on the local oxygen concentration while EPR imaging methods provide a spatial distribution of the trityl EPR signal, forming a diagnostic map of the local environment that is useful for treatment^3,15^.

While the narrow linewidth of trityl probes improves resolution for oximetry, narrow linewidths are typically indicative of slow relaxation and can lead to saturation effects using conventional EPR methods^27^. Trityl radical relaxation times are on the order of microseconds, a regime where pulse EPR methods have proven more advantageous; however, rapid scan (RS) EPR has been shown to produce the greatest increase in signal-to-noise ratios (SNR) relative to conventional continuous wave (CW) methods^28,29^. This improvement is primarily observed due to reduction of saturation effects, accomplished by adiabatic passage through resonance while subsequent deconvolution of the rapid passage effects allows undistorted narrow linewidths of these spin systems to be obtained^30–32^. Because SNR is highly dependent on resonator quality factor (Q), rapid scan experiments have typically been performed in high Q resonators while rapidly sweeping the microwave field using small sweep coils that offset the main magnetic field during the EPR experiment^30–32^. The rapid sweep rates used in rapid scan require broadband detection to accurately deconvolve rapid passage effects, which inversely affects resonator Q and may reduce SNR or introduce distortions in lineshape^33^. Because the aqueous environment typically utilized for trityl measurements creates an ideal environment for broadband measurements due to dielectric effects leading to microwave absorption, rapid scan is an effective method for trityl EPR oximetry measurements^34^. In other cases the resonator Q must be lowered significantly to obtain undistorted spectra, often through complicated techniques which are not compatible with *operando* experiments^35,36^.

**Fig. 2:**
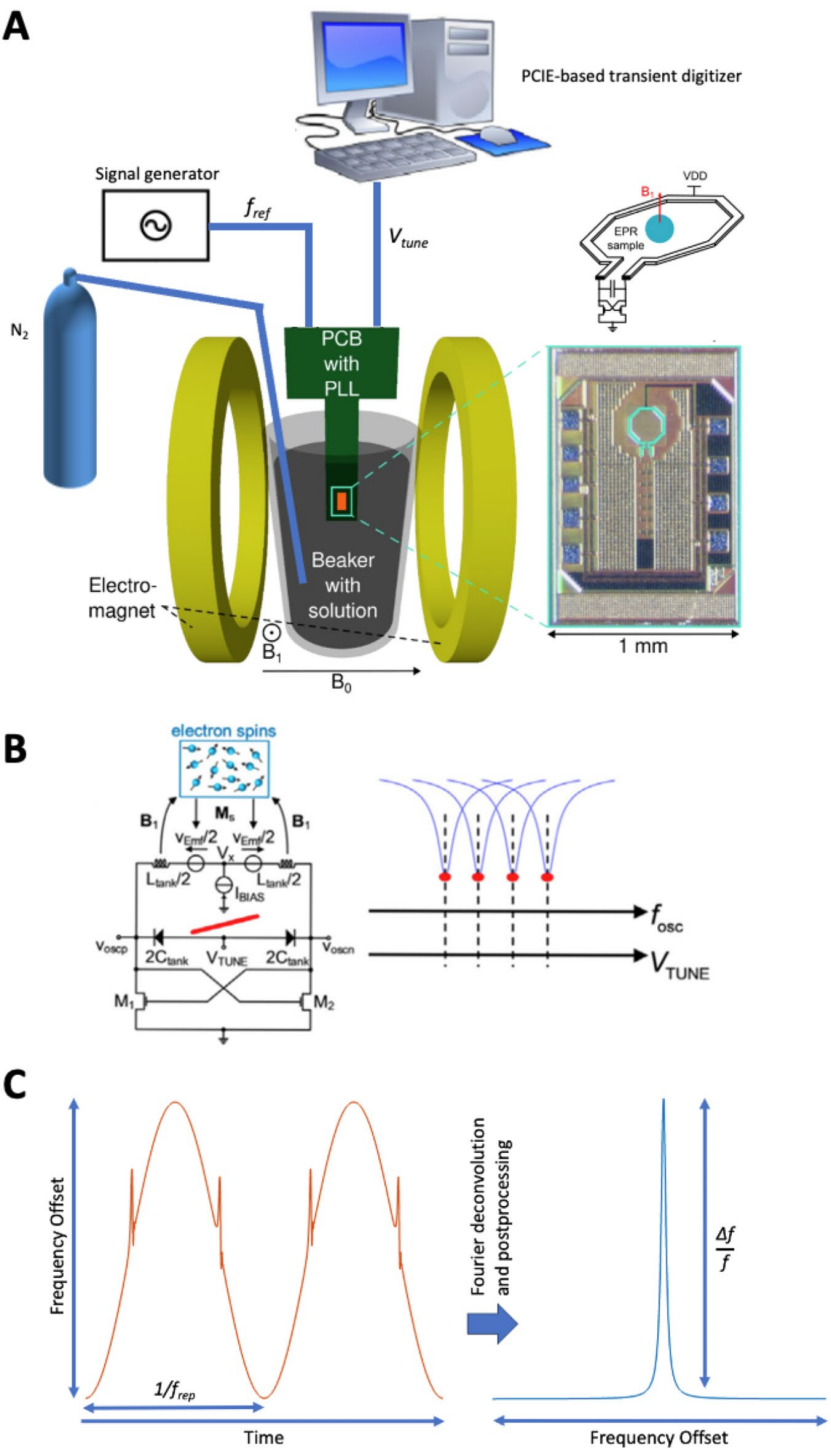
(**a**) An overview of the instrument configuration used for the experiments concerning oxygen detection using the EPRoC dipstick. An external oscillator provides the RF reference signal for the PLL. The single coil (diameter 200 $\:{\upmu\:}\text{m}$) EPRoC was coated with a 10–12 μm thick layer of parylene C and submerged into the solution. The RS data were recorded using the digitizer (**b**) Operating principle of the EPRoC showing the bi-directional coupling of the VCO to the electron spins. Changing the reference oscillator frequency results in a change of the VCO oscillation frequency via the varactor inside the VCO while the cross-coupled transistor pair provides a negative resistance. This acts to mitigate loss in the coil and loss while maintaining a pseudo-high Q circuit at every oscillation frequency. (**c**) The detection scheme utilized for frequency-swept, frequency-modulated rapid scan detection. The periodic signal is recorded in the time domain by sinusoidally sweeping the VCO with an amplitude *f*_*dev*_ and repetition rate *f*_*rep*_. The resulting periodic spin signal is Fourier deconvolved to recover the slow scan signal, detected as a spin-induced change in oscillator frequency, *Δf/f*, as the VCO is swept through resonance. Adapted from Refs^40,47,62^.

EPR-on-a-chip (EPRoC) devices have recently been demonstrated to perform EPR spectroscopy without the use of an EPR resonator by performing both excitation and detection of the spin signal response using voltage-controlled oscillators (VCOs)^37–41^. In this application, the cavity resonator is replaced by a single coil inductor and varactor, which function primarily in a similar fashion as the inductive-capacitive (LC) circuitry of a typical resonator (Fig. 2a). However, this lumped LC resonator may be interfaced to a cross coupled transistor pair providing a negative resistance capable of replenishing the energy lost in the LC resonator. While the primary purpose of this is to offset the conductive loss in the coil, losses due to high dielectric constant materials that absorb microwaves, which are significantly smaller than the overall coil loss, are also replenished^40,42–44^. By embedding the VCO in a phase-locked loop (PLL) circuit, its phase and frequency may be controlled both precisely and with very short response time by varying the reference frequency to create a wide (up to GHz) frequency sweep range for EPR spectroscopy^45^. The pseudo-high Q broadband circuit is facilitated by changing the oscillation frequency of the coil directly via manipulation of the varactor inside the VCO, which is controlled through the PLL by the reference frequency provided by a radio-frequency generator (Fig. 2b)^46^. The EPRoC is therefore ideal for detection of trityl radicals in the aqueous environment used for oximetry measurements. When properly isolated from the solvent via a protective coating (parylene C), the EPRoC may be used as a dipstick sensor for detection of free radicals directly in solution^47^.

Though the EPRoC has previously been demonstrated to perform frequency-swept rapid scan EPR with significantly improved SNR relative to CW EPR, slowly relaxing spin centers still pose a challenge for the EPRoC due the inherently high microwave excitation (B_1_), primarily due to the required bias current to produce stable oscillations in the small (200 μm) VCO^48–50^. Therefore, CW spectra recorded using the high intensity B_1_ of the EPRoC may exhibit saturation induced line broadening and lineshape distortions^50^. For this reason, a detection scheme similar to dispersion EPR has been developed via frequency-swept, frequency modulated rapid scan (Fig. 2c)^51–54^. In this way, the typical saturation of the absorption lineshape, obtained via amplitude modulation of the VCO, is circumvented both by recording the dispersion-like lineshape via frequency modulation, and by utilizing the increased B_1_ possible due to rapid passage through resonance with rapid scan^48,55,56^. Coupled with the fast digitization possible with rapid scan, it is feasible to accurately record the trityl lineshape with respect to dissolved oxygen concentration in fluid solution on a timescale relevant for real-time monitoring^48^.

Combining these methodologies in a single operational device, we herein present *operando* and in situ assessment of oxygen concentration using the well-characterized trityl radical probe, Ox071, in fluid solution using a frequency-swept, frequency modulated rapid scan EPRoC dipstick device. By submerging the EPRoC directly in the reaction solution, EPR spectra are obtained that provide adequate lineshape resolution and sufficient SNR to correlate the observed linewidths with the time-dependent concentration of dissolved oxygen directly in solution without additional modification to the sample environment.

## Materials and methods

### Sample solutions

The trityl probe molecule, Ox071, was synthesized as reported previously^57,58^. Upon successful synthesis, the spin probe was dissolved in a dilute phosphate-buffered saline solution containing 150 mM sodium chloride, 3 mM potassium chloride, 10 mM disodium hydrogen phosphate dihydrate, and 2 mM potassium phosphate (PBS Buffer pH 7.6 -10x conc., Jena Bioscience) to a final concentration of approximately 0.3 mM, which is comparable to previously reported clinical applications^17,22^.

### Instrument configuration

For RS-EPRoC experiments, the trityl solution was placed in a 20 ml beaker positioned in the center of a Bruker B-E 25 electromagnet. The EPRoC printed circuit board (PCB) was attached to a supporting rod which provided an interface to a stepper motor (Thorlabs PT3-Z8) that allows the lowering and lifting of the EPRoC PCB into and out of the solution. The design principles of EPRoC devices have been presented extensively elsewhere but are briefly described here for convenience^37–40,42–48,50,59^. The EPRoC chip contains a VCO oscillating at a frequency of around 13.44 GHz and a divide-by-32 frequency divider, the output of which is fed into an on-chip buffer used to embed the VCO in a phase-locked loop located on the PCB. To provide the reference frequency (f_ref_) for the PLL of the EPRoC, a signal generator (Rohde & Schwarz SMB100B) operating at 420 MHz was used. The sinusoidal modulation was applied directly to the reference frequency such that a frequency deviation (*f*_*dev*_) of 83.2 MHz was obtained at a repetition rate (*f*_*rep*_) of 35 kHz resulting in a scan rate of 18.3 THz/s, equivalent to 6.5 MG/s field sweeps. The required signal bandwidth for unattenuated detection considering the narrow linewidth of the Ox071 was between 18 and 60 MHz while the bandwidth of the EPRoC PLL for rapid scan detection was 5 MHz (see *Supp*. Fig S1). The 5 MHz bandwidth is limited by the parasitic capacitance and inductance present on the PCB traces. There have been other EPRoC designs which integrate the entire PLL circuit on-chip and have hence achieved much larger bandwidths up to 200 MHz^49,60,61^. Because of a voltage offset present in the EPRoC FM signal, a 20 dB AC coupled amplifier (Mini-Circuits Model ZFL-500LN) with a 20 dB attenuator placed on the input for a total zero gain amplification which allowed the signal intensity to be within the dynamic range of the transient digitizer (ADQ7DC, Teledyne SP Devices). The rapid scan signal was recorded using a sampling rate of 5 GS/s. A bias current (4 mA) defining the magnitude of B_1_ was supplied to the EPRoC PCB by a low noise current source (Keithley 6221). Dry nitrogen was flowed through the trityl solution by way of a flexible tube submerged directly into the trityl solution. Evacuation of oxygen was continued for $\:\approx\:6$ hours using relatively slow flow rates as to not alter the volume of the solution. Before beginning measurements, the flow of nitrogen was removed to further reduce the likelihood of concentration fluctuations during EPR measurements. The reoxygenation of the trityl solution was then monitored continuously by recording a RS-EPRoC spectrum at 30 s intervals. The acquisition time for each spectrum was 19 s and consisted of 70,000 accumulations, each containing seven frequency-swept spectra which were then summed during post-processing.

### Analysis of Operando measurements

Rapid scan measurements were performed by submersing the EPRoC in the Ox071 solution, followed by removal of oxygen via dry nitrogen gas to establish the minimum obtainable linewidth. Spectral acquisition parameters were optimized to reduce total acquisition time while maintaining sufficient SNR for accurate linewidth measurements. After six hours of purging with dry nitrogen gas, the Ox071 solution EPR spectrum was recorded sequentially during the reoxygenation process by acquiring a rapid scan spectrum every 30 s. Each spectrum was then baseline corrected to remove the sinusoidal background typical of rapid scan experiments using a composite sinusoidal fit and subsequent least-squares analysis before subtraction from the directly digitized frequency-swept spectra. The baseline corrected spectra were then separated by direction of the sweep, either via increasing frequency or decreasing frequency, and summed before being separately Fourier deconvolved to derive the resulting EPR signal^48,63^. The dispersion-like lineshapes were then Hilbert transformed using the Kramers-Kronig relation to obtain absorption-like lineshapes in order to be comparable with CW experiments performed using a conventional spectrometer (see *Supp*.) and with prior results reported in the literature using Ox071^64^.

The evolution of the linewidth over time during the reoxygenation process was monitored by fitting each absorption spectrum with a Lorentzian function,

1 $$\:y=\frac{2}{\pi\:\sqrt{3}}\times\:\frac{1}{{\Gamma\:}}\times\:\frac{1}{1+\frac{4}{3}\:{\left(\frac{x-{B}_{0}}{{\Gamma\:}}\right)}^{2}}$$

where $\:{\Gamma\:}$ is the distance between the inflection points and $\:{\text{B}}_{0}$ is the resonance value which have been estimated by least-square minimization using the *esfit* function included in the software package EasySpin 6 (https://easyspin.org)^65^. The values of $\:{\Gamma\:}$ have been plotted with respect to time to determine the rate at which the linewidth of the trityl signal was broadened by increasing oxygen concentration. This was achieved by fitting via an exponential function,

2 $$\:{\Gamma\:}\left(\text{t}\right)=-A\text{exp}\left(-\alpha\:\bullet\:t\right)+c$$

where $\:{\Gamma\:}\left(\text{t}\right)$ is the linewidth obtained at time *t*, $\:\alpha\:$ is the rate constant of the reoxygenation process, *t* is the time, *A* is the amplitude of the exponential change and *c* is the equilibrium value after the complete reoxygenation to atmospheric conditions. The time constant of the reoxygenation was calculated from the fit using the following relation, $\:\tau\:={\alpha\:}^{-1}$.

### Estimation of the partial pressure of oxygen

To estimate the partial pressure of oxygen, the relationship between the partial pressure of oxygen in solution and the linewidth of Ox071 (2 mM) recorded via free induction decay (FID) detected 2D-phantom EPR imaging at 300 MHz from Ref^15^ was modified to yield the following relationship,

3 $$\:p{O}_{2}\:=\frac{{\Gamma\:}\left(\text{t}\right)\:-q-n}{\sqrt{3}\:m}$$

where *q* is the linear offset obtained, *n* is a concentration broadening parameter often referred to as self-broadening and *m* is the angular coefficient reported in Ref^15^. Due to differences between the methods used to record the EPR spectrum reported in Ref^15^, where FID detection was used, and the methods reported herein, where instead the Hilbert transformed data were used. Therefore to compare the minimum linewidth observed in the herein reported experiments (50 mG) and those reported in Ref^15^ (90 mG), the partial pressure of oxygen differs by a factor of the square root of three and is necessary due to the inclusion of this factor in Eq. 1 to be comparable with data recorded by CW (see *Supp.*).

## Results

**Fig. 3:**
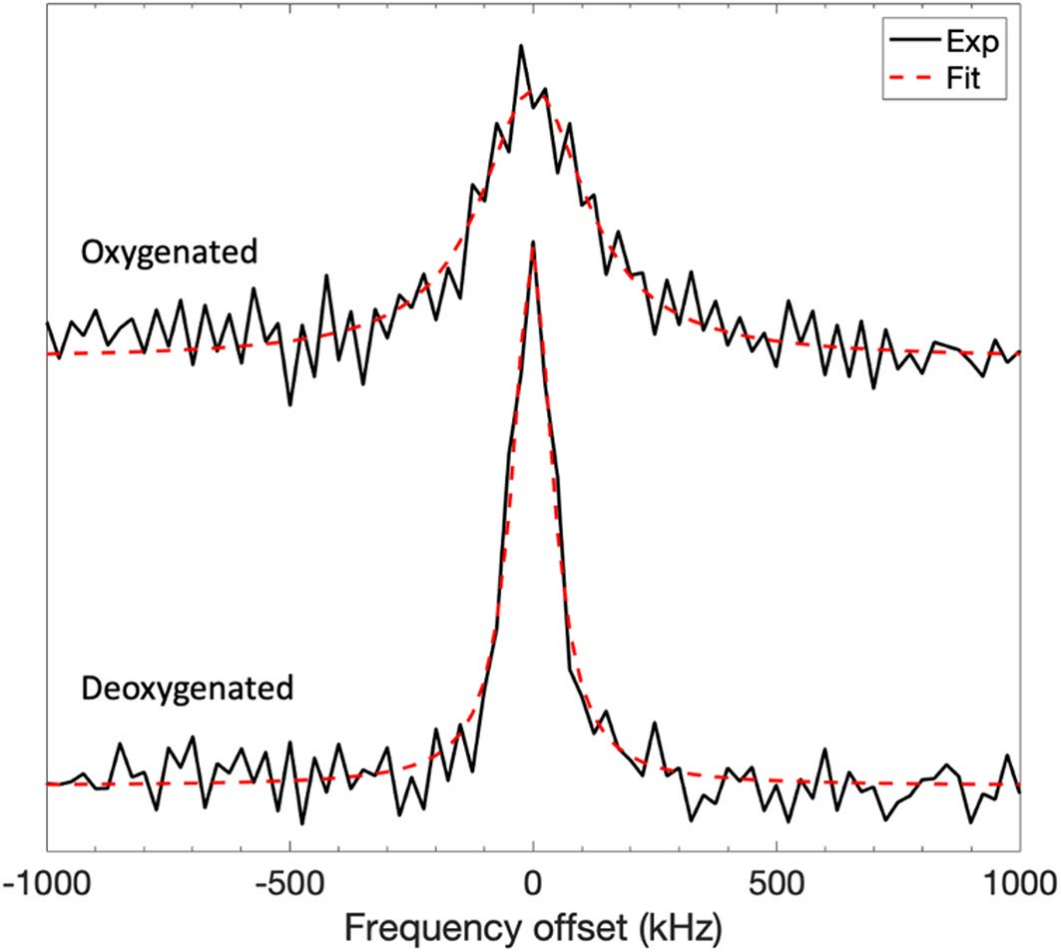
EPR spectra obtained via Hilbert transformation of the frequency-swept, frequency modulated rapid scan spectra obtained with the EPRoC for the trityl radical investigated, Ox071, shown both before and after evacuation of oxygen by purging with nitrogen gas. Fits to the data obtained by Lorentzian fitting are overlaid on the appropriate spectrum.

Frequency-swept, frequency-modulated rapid scan EPR measurements were performed using the EPRoC dipstick device on the trityl-containing solutions in presence of oxygen and after $\:\approx\:6$ hours of purging the solutions with dry-nitrogen gas. The spectra obtained, from which the linewidths were determined by *Lorentzian* fitting, are shown in Fig. 3. For the Ox071 solution, the oxygenated linewidth observed was 160 mG and the deoxygenated linewidth was 50 mG.

**Fig. 4:**
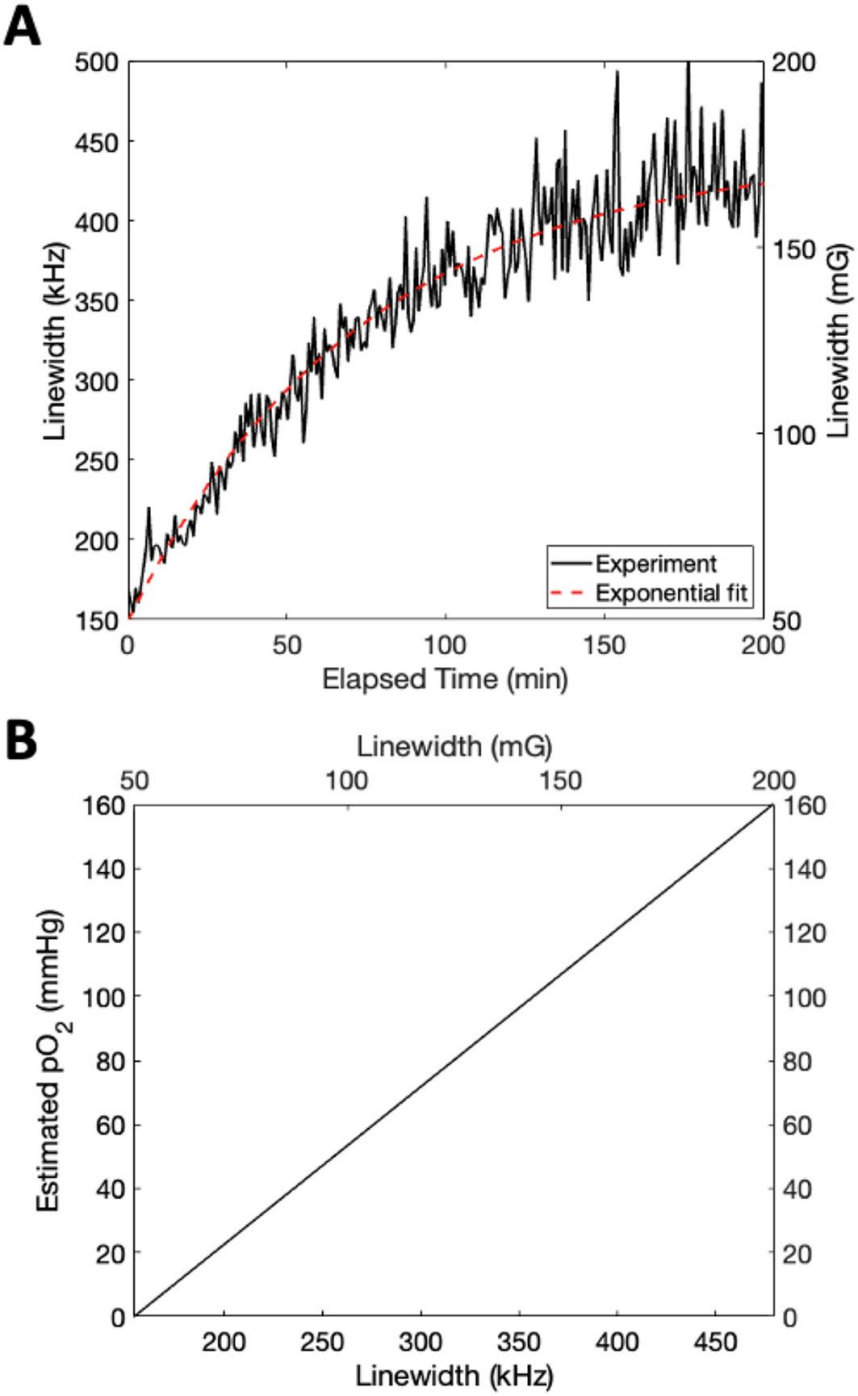
(**a**) The linewidth of the resulting EPR spectrum for each trityl as a function of reoxygenation time after stopping the flow of nitrogen through the solution via frequency-swept, frequency modulated rapid scan using the EPRoC sensor. The standard deviation of the measurements after reaching equilibrium was approximately 30 kHz. (**b**) The linewidth vs. oxygen (partial pressure) obtained via frequency-swept, frequency modulated rapid scan EPR using the dipstick EPRoC sensor via Eq. 3 using q = 7.637 mG, m = 0.1257 mG/mmHg, and *n* = 0.1726 mG.

To determine the potential to monitor reoxygenation of the trityl solution with respect to time, multiple frequency-swept, frequency-modulated rapid scan transients were continuously recorded after evacuation of oxygen using a continuous flow of dry nitrogen gas bubbled through the solution. Once a minimum linewidth in the deconvolved spectra was obtained, the nitrogen flow was stopped and the rapid scan signal was recorded. The reoxygenation was treated as an exponential process, and the fit to the linewidths obtained with respect to time for each sample is shown in Fig. 4.

Using the deoxygenated linewidth in the rapid scan experiments as a calibration factor, the approximate partial pressure of oxygen was obtained for the data shown in Fig. 4 using the relationship described in Eq. 3. A linear relationship may be obtained utilizing all linewidths recorded between the minimum and maximum linewidths observed. After inclusion of all consideration necessary for comparison due to discrepancies between data collection methods, the change in partial pressure of oxygen with respect to linewidth using the EPRoC sensor was found to be 0.53 mmHg O_2_ per kHz, equivalent to 1.47 mmHg O_2_ per mG. The detectable range observed using the EPRoC device is therefore approximately between zero and 140 mmHg O_2_.

## Discussion

In comparison to linewidths obtained via CW for trityl probes reported (see *Supp.* Fig S2), the linewidths observed in RS-EPRoC experiments demonstrate that the EPRoC sensor was successfully employed to detect dissolved oxygen over the range obtainable by nitrogen evacuation in aqueous environments which are typically problematic for EPR measurements at these frequencies (X-band) due to microwave absorption of the solvent^66^. Here, the VCO circuit is largely immune to dielectric loss from aqueous solvents, creating a stable B_1_ field throughout all measurements due to the negative resistance provided by the active pump circuit via the cross-coupled transistor pair and the comparatively lower E_1_ fields present relative to a typical cavity resonator^40,42–44^. It was observed in the experiments performed that the degree of saturation of the trityl systems was low enough to permit Kramers-Kronig transformations to an absorption-like lineshape (see *Supp*.); however, correction of the detected signal by phase manipulation was not possible due to the lack of quadrature detection^67^. Even with a presumed admixture of absorption and dispersion and the saturation observed, a calibration may be performed using solutions with known partial pressures of oxygen to accurately allow the EPRoC to function as an oxygen sensor.

Based on prior experiments, the expected linewidth change is approximately 1.45 mmHg O_2_ per mG while the EPRoC device reports 0.53 mmHg O_2_ per kHz, equivalent to 1.47 mmHg O_2_ per mG using Ox071 which is in considerably good agreement^15^. The experiments were performed with the solution open to the environment without additional modifications. The deoxygenated values are in agreement with prior reports, on the order of 50 mG; however, additional broadening was observed as the reoxygenation of the solution was recorded. It has been demonstrated in prior work that the VCO generates a substantial amount of heat during operation due to the power consumption of the device in a relatively small area (~ 30 mW/mm^2^) and this is the likely cause for the broadening at higher oxygen concentrations^68^. Because the N_2_ flow is removed before beginning to record the slow reoxygenation of such a large sample volume, there is no longer any method for removing the heat generated by the coils and therefore this significant heat generation likely causes the temperature of the water near the VCO to rise significantly. The heat produced cannot be easily removed or dissipated without an additional cooling mechanism. Because this is a reproducible phenomenon, the accuracy of the device in an oxygen-sensing environment is likely unaffected; however, the sensitivity suffers and is evidenced by the increased variability in the later measurements.

While field swept rapid scan imposes significant challenges associated with the increasing of scan rate due to the high voltages ($\:\approx\:$kV) required for rapid field sweeps, the frequency-swept, FM detection scheme circumvents these limitations because only the microwave frequency is swept^69,70^. This not only allows access to much wider sweep widths, but also allows for much faster scan rates as reported in ref^48^. One major drawback to frequency-swept, FM-detected rapid scan is the integration of noise over the entire frequency sweep (83.2 MHz). While the resonator acts as a bandwidth limitation in resonator-based rapid scan, it also acts as an effective noise filter that rejects all frequencies outside the bandwidth of the resonator (see *Supp.* Fig S3)^71^. Field-swept rapid scan inherently generates less noise in the acquired spectrum because of this; however, given the high resolution with which transient data may be recorded, digital filters with wide window functions can be applied to the recorded spectra in post-processing to improve the SNR. The standard deviation of the reoxygenated spectra after reaching equilibrium (~30 kHz) is primarily the result of a large inherent noise present when using VCOs and may be improved by increasing the number of VCOs in the array. This concept is further described in the supplementary information provided. The standard deviation reported here was calculated after reaching equilibrium, where the SNR for trityl measurements is lowest; therefore, this is the maximum observable standard deviation and is thereby reduced when lower oxygen concentrations are present, improving reproducibility. This may be further improved using higher trityl concentrations which may then be tailored to the range of oxygen concentration values of interest after consideration of concentration-dependent broadening effects.

When considering prior rapid scan experiments using EPRoC devices, it is possible to perform frequency-swept, AM-detected rapid scan with significant SNR improvements over CW-EPR within the same timescale; however, in the current design of the EPRoC devices, AM-detection is limited to a single coil^48^. Injection-locking has been recently demonstrated as a viable method to increase the sensitivity of EPRoC devices in a way that multiple coils may be used to produce a global phase-coherent microwave excitation field^43^. This reduces the complexity of a multicoil device by allowing the spin-induced frequency shift of the VCOs to be detected as a single FM output signal^40,48^.

One of the unique benefits of frequency-swept, frequency modulated rapid scan experiments is the alleviation of excitation bandwidth constraints imposed by the resonator in resonator-based rapid scan experiments. Because the frequency is swept at a rate that is rapid relative to spin diffusion, the entire sweep range of the VCO may be utilized for spin-excitation without distorting the spin response. In this way the detection system becomes the limiting bandwidth while the spin system is homogeneously excited, even if the PLL bandwidth limits the SNR of the detection due to filtering, but not distortion, of the high-frequency components of the rapid scan signal^48^. This bandwidth limit imposes a cutoff frequency beyond which the signal intensity is attenuated; however, the spin response is not altered and the lineshape is free of distortions induced by incomplete or nonuniform excitation due to limited resonator-bandwidth.

In both resonator-based EPR and in frequency-swept, FM-detected rapid scan, the fundamental spin sensitivity is defined by the sample with respect to number of spins comprising the total linewidth. In resonator-based CW-EPR the sensitivity is also defined by the Q factor of the resonator, the filling factor of the resonator, and the signal averaging rate which is determined by the digitizer and the ramp rate of the electromagnet^72^. In frequency-swept, FM-detected rapid scan the sensitivity does not suffer from low Q observed with solvent systems; and, given the design of the dipstick sensor, the filling factor is improved by way of submerging the detector in an apparent large volume relative to the detector coil diameter. Though less than half of the effective volume is utilized in the current design, EPRoCs compatible with microfluidics measurements improve upon this concept and may be incorporated into future fully-submersible applications^47,61,73^. While the sensitivity suffers in the herein reported rapid scan experiments due to the low repetition rate of the frequency sweep, this can be alleviated by utilizing higher bandwidth (on-chip) PLL circuits, like those that have successfully been demonstrated in pulse-EPRoC applications^49,61^.

## Conclusions and outlook

In this report we have demonstrated the *operando* and in situ capabilities of a submersible EPR-on-a-chip device capable of monitoring the concentration of dissolved oxygen using the trityl spin probe, Ox071. By using frequency-swept frequency-modulated rapid scan EPR methods, the limits on excitation bandwidth have been effectively circumvented resulting in EPR spectra that may be recorded on a timescale relevant for concentration monitoring in real-time. Similarly, the scalability of EPRoC and VCO based rapid scan techniques has been expanded by incorporation of the frequency-modulated EPRoC signal, which may be recorded in multiple coils of a VCO array, thereby removing both the volume limitations imposed by the small volume of a single coil and the scaling limitations imposed by amplitude-modulated rapid scan detection in EPRoC devices. The methods reported herein while applied to the application for oximetry may be generally applied to any spin system of interest that falls within the bandwidth of the EPRoC device; and, when narrow linewidths are not necessary, may also be applied in applications where a lower-homogeneity permanent magnet might be used. Because of the scalability of EPRoC devices, new geometries, increased coil diameters (to several mm), and even distributions of EPRoC VCO arrays may be considered, such as incorporation into a needle or other surgical devices to establish a spatial component while also considering mesh arrays or even more complicated geometries for surface-accessible diagnostics^22,74^. All of these advancements will allow the EPRoC to continue to progress and evolve as a point-of-care technology.

## Electronic supplementary material

Below is the link to the electronic supplementary material.

Supplementary Material 1

## Data Availability

The datasets used and/or analyzed during the current study are available from the corresponding author on reasonable request.

## References

[1] Brustolon, M. & Giamello, E. (eds) *Electron Paramagnetic Resonance: A Practitioner’s Toolkit* (Wiley, 2009). 10.1002/9780470432235.

[2] Liu, S., Timmins, G. S., Shi, H., Gasparovic, C. M. & Liu, K. J. Application of *in vivo* EPR in brain research: monitoring tissue oxygenation, blood flow, and oxidative stress. *NMR Biomed.* **17** (5), 327–334. 10.1002/nbm.899 (2004). 15366032 10.1002/nbm.899

[3] Ahmad, R., Kuppusamy, P. & Theory Instrumentation, and applications of electron paramagnetic resonance oximetry. *Chem. Rev.* **110** (5), 3212–3236. 10.1021/cr900396q (2010). 20218670 10.1021/cr900396q PMC2868962

[4] Swartz, H. M. et al. Clinical EPR: unique opportunities and some challenges. *Acad. Radiol.* **21** (2), 197–206. 10.1016/j.acra.2013.10.011 (2014). 24439333 10.1016/j.acra.2013.10.011 PMC3921887

[5] Halliwell, B. & Gutteridge, J. M. C. *Free Radicals in Biology and Medicine* (Oxford University Press, 2015). 10.1093/acprof:oso/9780198717478.001.0001.

[6] Donatienne, H. et al. A versatile EPR toolbox for the simultaneous measurement of oxygen consumption and superoxide production. *Redox Biol.* **40**, 101852. 10.1016/j.redox.2020.101852 (2021). 33418140 10.1016/j.redox.2020.101852 PMC7804984

[7] Mariappan, N., Elks, C. M., Fink, B., Francis, J. & TNF-Induced Mitochondrial Damage: A link between mitochondrial complex I activity and left ventricular dysfunction. *Free Radic Biol. Med.* **46** (4), 462–470. 10.1016/j.freeradbiomed.2008.10.049 (2009). 19041937 10.1016/j.freeradbiomed.2008.10.049 PMC2735225

[8] Martin, R. M. et al. Toward a nanoencapsulated EPR imaging agent for clinical use. *Mol. Imaging Biol.*10.1007/s11307-023-01863-0 (2023). 37870648 10.1007/s11307-023-01863-0 PMC11035482

[9] Gallez, B. et al. Small particles of fusinite and carbohydrate Chars coated with aqueous soluble polymers: Preparation and applications for *in vivo* EPR oximetry. *Magn. Reson. Med.* **40** (1), 152–159. 10.1002/mrm.1910400120 (1998). 9660565 10.1002/mrm.1910400120

[10] Ilangovan, G., Li, H., Zweier, J. L. & Kuppusamy, P. Electrochemical Preparation and EPR Studies of Lithium Phthalocyanine. 3. Measurements of Oxygen Concentration in Tissues and Biochemical Reactions. 10.1021/jp010130+ (2001).

[11] Zweier, J. L., Chzhan, M., Ewert, U., Schneider, G. & Kuppusamy, P. Development of a highly sensitive probe for measuring oxygen in biological tissues. *J. Magn. Reson. B*. **105** (1), 52–57. 10.1006/jmrb.1994.1099 (1994). 7921671 10.1006/jmrb.1994.1099

[12] Bobko, A. A. et al. Trityl-Based EPR probe with enhanced sensitivity to oxygen. *Free Radic Biol. Med.* **47** (5), 654–658. 10.1016/j.freeradbiomed.2009.06.007 (2009). 19523513 10.1016/j.freeradbiomed.2009.06.007 PMC2739013

[13] Khramtsov, V. V. In Vivo spectroscopy and imaging of nitroxide probes. In Nitroxides - Theory, Experiment and Applications; InTech, 10.5772/39129. (2012).

[14] Dunn, J. F. & Swartz, H. M. In vivo electron paramagnetic resonance oximetry with particulate materials. *Methods* **30** (2), 159–166. 10.1016/S1046-2023(03)00077-X (2003). 12725782 10.1016/s1046-2023(03)00077-x

[15] Matsumoto, K. et al. EPR-based oximetric imaging: A combination of single Point‐based Spatial encoding and T1 weighting. *Magn. Reson. Med.* **80** (5), 2275–2287. 10.1002/mrm.27182 (2018). 29582458 10.1002/mrm.27182 PMC8080971

[16] Gertsenshteyn, I. et al. Absolute Oxygen-Guided Radiation Therapy Improves Tumor Control in Three Preclinical Tumor Models. *Front Med (Lausanne)* **10**. 10.3389/fmed.2023.1269689 (2023). 10.3389/fmed.2023.1269689 PMC10613495 37904839

[17] Epel, B., Viswakarma, N., Sundramoorthy, S. V., Pawar, N. J. & Kotecha, M. Oxygen imaging of a rabbit tumor using a Human-Sized pulse electron paramagnetic resonance imager. *Mol. Imaging Biol.*10.1007/s11307-023-01852-3 (2023). 37715089 10.1007/s11307-023-01852-3

[18] Walsh, J. C. et al. The clinical importance of assessing tumor hypoxia: relationship of tumor hypoxia to prognosis and therapeutic opportunities. *Antioxid. Redox Signal.* **21** (10), 1516–1554. 10.1089/ars.2013.5378 (2014). 24512032 10.1089/ars.2013.5378 PMC4159937

[19] Schaner, P. E. et al. OxyChip Implantation and Subsequent Electron Paramagnetic Resonance Oximetry in Human Tumors Is Safe and Feasible: First Experience in 24 Patients. *Front. Oncol.*. **10**. 10.3389/fonc.2020.572060 (2020). 10.3389/fonc.2020.572060 PMC7653093 33194670

[20] Epel, B., Redler, G. & Halpern, H. J. *Oxygen Transport to Tissue XXXVI*; Swartz, H. M., Harrison, D. K., Bruley, D. F., Eds.; Advances in Experimental Medicine and Biology; Springer New York: New York, NY, Vol. 812. 10.1007/978-1-4939-0620-8 (2014). 10.1007/978-1-4939-0620-8_49 PMC7058375 29435858

[21] Ruigrok, M. J. R. et al. The effects of oxygen concentration on cell death, Anti-Oxidant transcription, acute inflammation, and cell proliferation in Precision-Cut lung slices. *Sci. Rep.* **9** (1), 16239. 10.1038/s41598-019-52813-2 (2019). 31700101 10.1038/s41598-019-52813-2 PMC6838147

[22] Hameed, S. et al. Longitudinal, 3D oxygen imaging of cells in a Multi-Well plate using pulse electron paramagnetic resonance imaging. *Npj Imaging*. **2** (1), 8. 10.1038/s44303-024-00013-7 (2024). 10.1038/s44303-024-00013-7 PMC12118754 40604125

[23] Ardenkjær-Larsen, J. H. et al. EPR and DNP properties of certain novel single electron contrast agents intended for oximetric imaging. *J. Magn. Reson.* **133** (1), 1–12. 10.1006/jmre.1998.1438 (1998). 9654463 10.1006/jmre.1998.1438

[24] Yong, L. et al. Electron spin relaxation of triarylmethyl radicals in fluid solution. *J. Magn. Reson.* **152** (1), 156–161. 10.1006/jmre.2001.2379 (2001). 11531374 10.1006/jmre.2001.2379

[25] Subramanian, S. et al. Noninvasive in vivo oximetric imaging by radiofrequency FT EPR. *Magn. Reson. Med.* **47** (5), 1001–1008. 10.1002/mrm.10133 (2002). 11979580 10.1002/mrm.10133

[26] Williams, B. B. et al. Imaging spin probe distribution in the tumor of a living mouse with 250 mhz EPR: correlation with BOLD MRI. *Magn. Reson. Med.* **47** (4), 634–638. 10.1002/mrm.10089 (2002). 11948723 10.1002/mrm.10089

[27] Eaton, S. S. & Eaton, G. R. Relaxation mechanisms. *eMagRes* **5** (4), 1543–1556. 10.1002/9780470034590.emrstm1507 (2016).

[28] Owenius, R., Eaton, G. R. & Eaton, S. S. Frequency (250 MHz to 9.2 GHz) and viscosity dependence of electron spin relaxation of triarylmethyl radicals at room temperature. *J. Magn. Reson.* **172** (1), 168–175. 10.1016/j.jmr.2004.10.007 (2005). 15589420 10.1016/j.jmr.2004.10.007

[29] Mitchell, D. G. et al. X-Band Rapid-Scan EPR of samples with long electron spin relaxation times: A comparison of continuous wave, pulse and Rapid-Scan EPR. *Mol. Phys.* **111** (18–19), 2664–2673. 10.1080/00268976.2013.792959 (2013).

[30] Eaton, S. S. et al. Rapid-Scan EPR imaging. *J. Magn. Reson.* **280**, 140–148. 10.1016/j.jmr.2017.02.013 (2017). 28579099 10.1016/j.jmr.2017.02.013 PMC5523658

[31] Joshi, J. P. et al. Rapid-Scan EPR with triangular scans and fourier Deconvolution to recover the Slow-Scan spectrum. *J. Magn. Reson.* **175** (1), 44–51. 10.1016/j.jmr.2005.03.013 (2005). 15949747 10.1016/j.jmr.2005.03.013

[32] Tseitlin, M., Rinard, G. A., Quine, R. W., Eaton, S. S. & Eaton, G. R. Deconvolution of sinusoidal rapid EPR scans. *J. Magn. Reson.* **208** (2), 279–283. 10.1016/j.jmr.2010.11.015 (2011). 21163677 10.1016/j.jmr.2010.11.015 PMC3097533

[33] Eaton, G. R., Eaton, S. S. & Rapid-Scan *Electron. Paramagnetic Reson. eMagRes* **5** (4), 1529–1542. 10.1002/9780470034590.emrstm1522. (2016).

[34] Nesmelov, Y. E., Gopinath, A. & Thomas, D. D. Aqueous sample in an EPR cavity: sensitivity considerations. *J. Magn. Reson.* **167** (1), 138–146. 10.1016/j.jmr.2003.12.005 (2004). 14987608 10.1016/j.jmr.2003.12.005 PMC3804294

[35] Mitchell, D. G., Quine, R. W., Tseitlin, M., Eaton, S. S. & Eaton, G. R. X-Band Rapid-Scan EPR of nitroxyl radicals. *J. Magn. Reson.* **214**, 221–226. 10.1016/j.jmr.2011.11.007 (2012). 22169156 10.1016/j.jmr.2011.11.007

[36] Eaton, G. R. & Eaton, S. S. Advances in rapid scan EPR spectroscopy. In *Methods in Enzymology*; Academic Press Inc. Vol. 666, 1–24. 10.1016/bs.mie.2022.02.013 (2022). 10.1016/bs.mie.2022.02.013 PMC10093151 35465917

[37] Handwerker, J. et al. 28.2 A 14GHz Battery-Operated Point-of-Care ESR Spectrometer Based on a 0.13µm CMOS ASIC. In *IEEE International Solid-State Circuits Conference (ISSCC)*; IEEE, 2016; pp 476–477. 10.1109/ISSCC.2016.7418114 (2016).

[38] Yalcin, T. & Boero, G. Single-Chip detector for electron spin resonance spectroscopy. *Rev. Sci. Instrum.* **79** (9). 10.1063/1.2969657 (2008). 10.1063/1.2969657 19044436

[39] Schlecker, B. et al. VCO-Based ESR-on-a-Chip as a tool for Low-Cost, High-Sensitivity Point-of-Care diagnostics. In 2017 IEEE SENSORS; IEEE, 1–3. 10.1109/ICSENS.2017.8233896. (2017).

[40] Anders, J. & Lips, K. MR to go. *J. Magn. Reson.* **306**, 118–123. 10.1016/j.jmr.2019.07.007 (2019). 31327536 10.1016/j.jmr.2019.07.007

[41] Yang, X., Babakhani, A. A. & Single-Chip Electron paramagnetic resonance transceiver in 0.13-Μm SiGe BiCMOS. *IEEE Trans. Microw. Theory Tech.* **63** (11), 3727–3735. 10.1109/TMTT.2015.2481895 (2015).

[42] Razavi, B. The Cross-Coupled Pair - Part I [A circuit for all seasons]. *IEEE Solid-State Circuits Mag.* **6** (3), 7–10. 10.1109/MSSC.2014.2329234 (2014).

[43] Chu, A., Schlecker, B., Lips, K., Ortmanns, M. & Anders, J. An 8-Channel 13GHz ESR-on-a-Chip Injection-Locked Vco-Array Achieving 200µM-Concentration Sensitivity. In *IEEE International Solid - State Circuits Conference* - *(ISSCC)*; IEEE, 2018; pp 354–356. 10.1109/ISSCC.2018.8310330 (2018).

[44] Chu, A. et al. On the modeling of Amplitude-Sensitive electron spin resonance (ESR) detection using Voltage-Controlled oscillator (VCO)-Based ESR-on-a-Chip detectors. *Magn. Reson.* **2** (2), 699–713. 10.5194/mr-2-699-2021 (2021). 10.5194/mr-2-699-2021 PMC10539732 37905224

[45] Anders, J. Nonlinear modeling of Continuous-Wave spin detection using Oscillator-Based ESR-on-a-Chip sensors. In *Studies in systems, decision and control*. *Springer Int. Publishing*. **109**, 57–87. 10.1007/978-3-319-58996-1_4 (2018).

[46] Chu, A. et al. VCO-Based ESR-on-a-Chip as a Tool for Low-Cost, High-Sensitivity Food Quality Control. In *2017 IEEE Biomedical Circuits and Systems* Conference *(BioCAS)*; IEEE, ; pp 1–4 (2017). 10.1109/BIOCAS.2017.8325172.

[47] Künstner, S. et al. Monitoring the state of charge of vanadium redox flow batteries with an EPR-on-a-Chip dipstick sensor. *Phys. Chem. Chem. Phys.* **26** (25), 17785–17795. 10.1039/D4CP00373J (2024). 38874514 10.1039/d4cp00373j

[48] Künstner, S. et al. Rapid-Scan electron paramagnetic resonance using an EPR-on-a-Chip sensor. *Magn. Reson.* **2** (2), 673–687. 10.5194/mr-2-673-2021 (2021). 10.5194/mr-2-673-2021 PMC10539758 37905212

[49] Hassan, M. A. et al. A 14-Channel 7 GHz VCO-Based EPR-on-a-Chip Sensor with Rapid Scan Capabilities. In *2021 IEEE Sensors*; Vol. 2021-October, pp 1–4. 10.1109/SENSORS47087.2021.9639513 (2021).

[50] Künstner, S. et al. Microwave Field Mapping for EPR on a Chip Experiments. *Sci Adv Accepted*. (2024). 10.1126/sciadv.ado5467 PMC11801239 39151005

[51] Laguta, O., Tuček, M., van Slageren, J. & Neugebauer, P. Multi-Frequency Rapid-Scan HFEPR. *J. Magn. Reson.* **296**, 138–142. 10.1016/j.jmr.2018.09.005 (2018). 30261338 10.1016/j.jmr.2018.09.005

[52] Hyde, J. S., Strangeway, R. A., Camenisch, T. G., Ratke, J. J. & Froncisz, W. W. -Band Frequency-Swept EPR. *J. Magn. Reson.* **205** (1), 93–101. 10.1016/j.jmr.2010.04.005 (2010). 20462775 10.1016/j.jmr.2010.04.005 PMC2885579

[53] Hyde, J. S. et al. Microwave frequency modulation in CW EPR at W-Band using a Loop-Gap resonator. *J. Magn. Reson.* **185** (2), 259–263. 10.1016/j.jmr.2007.01.002 (2007). 17267251 10.1016/j.jmr.2007.01.002

[54] Segantini, M. et al. Compact Electron Paramagnetic Resonance on a Chip Spectrometer Using a Single Sided Permanent Magnet. *ACS Sens Submitted*. (2024). 10.1021/acssensors.4c00788 PMC11519922 39326012

[55] Hyde, J. S., Froncisz, W. & Kusumi, A. Dispersion electron spin resonance with the Loop-Gap resonator. *Rev. Sci. Instrum.* **53** (12), 1934–1937. 10.1063/1.1136918 (1982).

[56] Rinard, G. A., Quine, R. W., Ghim, B. T., Eaton, S. S. & Eaton, G. R. Dispersion and superheterodyne EPR using a bimodal resonator. *J. Magn. Reson. A*. **122** (1), 58–63. 10.1006/jmra.1996.0174 (1996).

[57] Poncelet, M., Huffman, J. L., Khramtsov, V. V., Dhimitruka, I. & Driesschaert, B. Synthesis of hydroxyethyl Tetrathiatriarylmethyl radicals OX063 and OX071. *RSC Adv.* **9** (60), 35073–35076. 10.1039/C9RA08633A (2019). 32483485 10.1039/c9ra08633a PMC7263632

[58] Poncelet, M. et al. Synthesis and characterization of a biocompatible 13C1 isotopologue of trityl radical OX071 for in vivo EPR viscometry. *Analyst* **147** (24), 5643–5648. 10.1039/D2AN01527G (2022). 36373434 10.1039/d2an01527g PMC9729415

[59] Anders, J., Fully-Integrated, C. M. O. S. & Lausanne Probes for Magnetic Resonance Applications, EPFL. 10.5075/epfl-thesis-5154 (2011).

[60] Khan, K. et al. A 12.2 to 14.9 GHz Injection-Locked VCO Array with an on-Chip 50 MHz BW Semi-Digital PLL for Transient Spin Manipulation and Detection. In *2022 IEEE 65th International Midwest Symposium on Circuits and Systems (MWSCAS)*; IEEE, ; Vol. 2022-August, pp 1–4. (2022). 10.1109/MWSCAS54063.2022.9859288.

[61] Hassan, M. A. et al. Towards Single-Cell pulsed EPR using VCO-Based EPR-on-a-Chip detectors. *Frequenz* **76**(11–12), 699–717. 10.1515/freq-2022-0096 (2022).

[62] Anders, J. et al. Progress in miniaturization and Low-Field nuclear magnetic resonance. *J. Magn. Reson.* **322**, 106860. 10.1016/j.jmr.2020.106860 (2021). 33423757 10.1016/j.jmr.2020.106860

[63] Stoner, J. W. et al. Direct-Detected Rapid-Scan EPR at 250 MHz. *J. Magn. Reson.* **170** (1), 127–135. 10.1016/j.jmr.2004.06.008 (2004). 15324766 10.1016/j.jmr.2004.06.008

[64] Portis, A. M. Electronic structure of F centers: saturation of the electron spin resonance. *Phys. Rev.* **91** (5), 1071–1078. 10.1103/PhysRev.91.1071 (1953).

[65] Stoll, S. & Schweiger, A. EasySpin, a comprehensive software package for spectral simulation and analysis in EPR. *J. Magn. Reson.* **178** (1), 42–55. 10.1016/j.jmr.2005.08.013 (2006). 16188474 10.1016/j.jmr.2005.08.013

[66] Dalal, D. P., Eaton, S. S. & Eaton, G. R. The Effects of Lossy Solvents on Quantitative EPR Studies. *J. Magn. Reson.* **44**(3), 415–428. 10.1016/0022-2364(81)90276-6 (1969).

[67] Tseitlin, M., Quine, R. W., Rinard, G. A., Eaton, S. S. & Eaton, G. R. Combining absorption and dispersion signals to improve Signal-to-Noise for Rapid-Scan EPR imaging. *J. Magn. Reson.* **203** (2), 305–310. 10.1016/j.jmr.2010.01.013 (2010). 20181505 10.1016/j.jmr.2010.01.013 PMC2856439

[68] Segantini, M. et al. Electrically detected magnetic resonance on a chip (EDMRoC) for analysis of Thin-Film silicon photovoltaics. *Magnetochemistry* **9** (7), 183. 10.3390/magnetochemistry9070183 (2023).

[69] Quine, R. W., Mitchell, D. G., Tseitlin, M., Eaton, S. S. & Eaton, G. R. A resonated coil driver for rapid scan EPR. *Concepts Magn. Reson. Part. B Magn. Reson. Eng.* **41B** (4), 95–110. 10.1002/cmr.b.21222 (2012).

[70] Quine, R. W., Czechowski, T. & Eaton, G. R. A linear magnetic field scan driver. *Concepts Magn. Reson. Part. B Magn. Reson. Eng.* **35B** (1), 44–58. 10.1002/cmr.b.20128 (2009). 19838315 10.1002/cmr.b.20128 PMC2762224

[71] Lin, Y. J., Tseng, Y. C. & Wu, T. L. A Resonator-Based suppressor for mitigating the noise transfer on metal plates for control of electromagnetic interference. *IEEE Microwave Wirel. Compon. Lett.* **26** (11), 906–908. 10.1109/LMWC.2016.2615002 (2016).

[72] Eaton, G. R., Eaton, S. S., Barr, D. P., Weber, R. T. & Quantitative, E. P. R. Springer Vienna: Vienna, (2010). 10.1007/978-3-211-92948-3

[73] Dayan, N. et al. Electron spin resonance microfluidics with subnanoliter liquid samples. *J. Magn. Reson. Open.* **2-3**, 100005. 10.1016/j.jmro.2020.100005 (2020).

[74] Handwerker, J. et al. A CMOS NMR needle for probing brain physiology with high Spatial and Temporal resolution. *Nat. Methods*. **17** (1), 64–67. 10.1038/s41592-019-0640-3 (2020). 31768059 10.1038/s41592-019-0640-3

