# Supplementary material for: Operando detection of dissolved oxygen in fluid solution using a submersible rapid scan EPR on a chip dipstick sensor
Source: Sci Rep. 2025 Mar 21;15:9872. doi: 10.1038/s41598-025-93591-4 (PMC11928686; doi:10.1038/s41598-025-93591-4)
Supplement: Supplementary file 1 — Supplementary Material 1 [file 41598_2025_93591_MOESM1_ESM.docx]

*Operando* detection of dissolved oxygen in fluid solution using a submersible rapid scan EPR on a chip dipstick sensor

Joseph E. McPeak^1,2^, Michele Segantini^1^, Gianluca Marcozzi^1^, Irene Dona^1^, Silvio Künstner^1^, Anh Chu^3^, Michal Kern^3^, Martin Poncelet^4,5^, Benoit Driesschaert^4,5^, Jens Anders^3,6^, and Klaus Lips^1,7^

^1^Berlin Joint EPR Laboratory and EPR4Energy, Department Spins in Energy Conversion and Quantum Information Science (ASPIN), Helmholtz-Zentrum Berlin für Materialien und Energie GmbH, Berlin, Germany

^2^Novo Nordisk Foundation Pulse EPR Center, Department of Chemistry, University of Copenhagen, Copenhagen, Denmark

^3^Institute of Smart Sensors, Universität Stuttgart, Stuttgart, Germany

^4^Department of Pharmaceutical Sciences, School of Pharmacy, West Virginia University, Morgantown, West Virginia, USA

^5^In Vivo Multifunctional Magnetic Resonance Center, Robert C. Byrd Health Sciences Center, West Virginia University, Morgantown, West Virginia, USA

^6^Center for Integrated Quantum Science and Technology (IQST), Stuttgart and Ulm, Germany

^7^Berlin Joint EPR Laboratory, Fachbereich Physik, Freie Universität Berlin, Berlin, Germany

Correspondence to: Joseph.mcpeak@helmholtz-berlin.de

**Supplementary information**

*EPRoC excitation vs. detection bandwidth*

For excitation, both the oscillation frequency and the resonance frequency of the LC tank inside the oscillator follow the tuning voltage with a very large bandwidth, which is essentially limited by parasitics of the varactor and routing parasitics. Since these parasitics are small in modern integrated circuit technologies, for the rapid scan experiments reported herein the dynamic range of the VCO can be modeled quasi-statically such that the change in oscillation frequency, $f_{\mathrm{VCO}}$, occurs instantaneously with a change in the tuning voltage, $V_{\mathrm{TUNE}}$, according to $f_{\mathrm{VCO}}=K_{\mathrm{VCO}}\left( V_{\mathrm{TUNE}} \right)\cdot V_{\mathrm{TUNE}}$, where $K_{\mathrm{VCO}}$ is the voltage-dependent VCO sensitivity which encompasses the nonlinearity of the VCO.^1^ Therefore, when embedding the VCO into a PLL, it is purely the PLL bandwidth which is limiting the excitation bandwidth of the EPRoC system.^1^

For detection, the transient spin magnetization induces an electromotive force (emf), $v_{\mathrm{emf}}(t)$, in the tank coil, which, in turn, modulates the oscillator frequency according to Ref^2^,

|  | $\delta f\approx-\frac{\omega_{\mathrm{osc}}}{A_{0}}\cdot v_{\mathrm{emf}}\left( t \right)\cdot\cos\left( \omega_{\mathrm{osc}}t \right)$ | Eq. S1 |
| --- | --- | --- |

where $\omega_{\mathrm{osc}}$ is the oscillation frequency (in rad/s) and $A_{0}$ is the oscillation amplitude. Therefore, the spin magnetization produces an inertia-free change of the oscillation frequency, which corresponds to a quasi-static detector characteristic. The physical explanation for this quasi-infinite bandwidth is that a change in oscillation frequency does not require a change in the energy stored in the tank. Naturally, there are again parasitics in the system that limit the VCO detection bandwidth to a finite, but still very large value. Consequently, the detection bandwidth is also not limited by the EPRoC detector but by the bandwidth of the PLL into which the VCO is embedded. Here, it should be noted that the EPR signal can, in principle, also be detected outside the PLL bandwidth as demonstrated herein and in Ref^3^.

*Calculation of signal bandwidth in rapid scan*

The calculation of the required signal bandwidth for accurate excitation and detection of rapid scan is given in Ref^4^ (Eq. 13) and has been adapted for frequency-swept rapid scan as follows,

|  | ${BW}_{signal}\approx N\alpha T_{2}^{*}=2\pi{Nf}_{rep}f_{dev}T_{2}^{*}$ | Eq. S2 |
| --- | --- | --- |

where $N$ is a coefficient of acceptable line broadening and is usually selected to be between 3-5, $\alpha$ is the scan rate, $f_{rep}$ is the repetition rate, $f_{dev}$ is the frequency deviation, and $T_{2}^{*}$ the transverse relaxation time.^4,5^ Rather than measure $T_{2}^{*}$ directly, the linewidth observed may be substituted, to now facilitate calculation of required signal bandwidth over the range of oxygen concentrations accessible as follows,

|  | ${BW}_{signal}\approx N\alpha T_{2}^{*}=2\pi Nf_{rep}f_{dev}(\frac{1}{\sqrt{3}\pi Lw})$ | Eq. S3 |
| --- | --- | --- |

where the observed linewidth, $Lw$, directly relates to the $T_{2}^{*}$ of the spin system.^6^ Using $f_{rep}$ of 35 kHz and a $f_{dev}$ of 83.2 MHz yields a scan rate of approximately 18.3 THz/s equivalent to 6.5 MG/s field sweeps. Using a broadening parameter of $N=5$, the bandwidths required are between 38 and 120 MHz for 160 and 50 mG linewidths, respectively, while the PLL bandwidth of the EPRoC device is only 5 MHz. The rapid scan signal recorded in the time domain under these conditions is shown in Fig S1. Here the transient or “wiggles” resulting from rapid passage through resonance are undistorted, demonstrating a decoupling of excitation bandwidth, which is limited by resonator bandwidth in field-swept, resonator based rapid scan experiments, and detection bandwidth, which is instead limited by PLL bandwidth in this EPRoC design.^4,6,7^ In the herein reported experiments, the entire spin packet is excited during the frequency sweep, without any filtering of the rapid scan transient response allowing undistorted signal acquisition such that only the attenuated signal response resulting from detection outside of the effective bandwidth of the PLL limits the recorded signal intensity. The simulation in Fig S1 was performed using the *blochsteady* function of Easyspin (ver. 5.2.35).^8^ The simulation utilizes the T_2_^*^ calculated from the observed linewidth of the deoxygenated spectrum, 1.3 microseconds, and agrees well with the recorded data until the intensity of the oscillations in the recorded data fall below the noise floor of the device. Additional parameters included in the simulation were as follows; g = 2.003, T_1_ = 10 microseconds, B_1_ = 0.0108 mT, modulation amplitude = 2.97 mT, modulation frequency = 35 kHz. A least squares analysis was not performed. The resulting simulation was scaled appropriately to be of equivalent intensity to the recorded data.


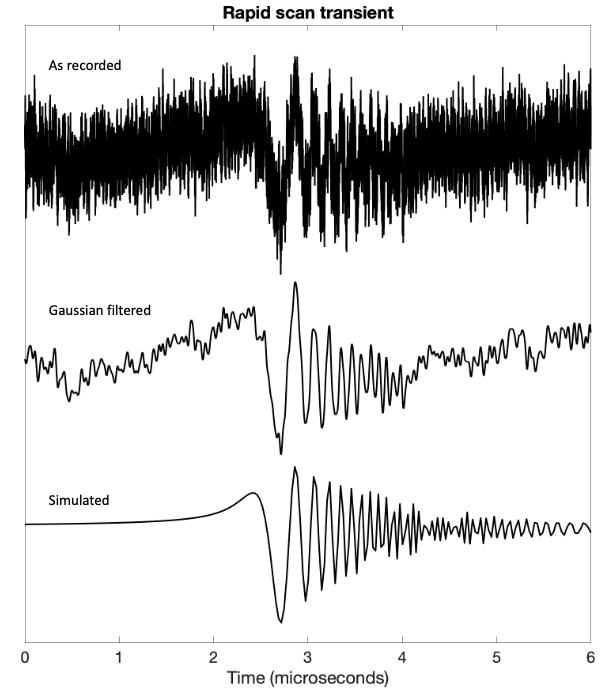


Figure S1: The transient rapid scan signal from Ox071 recorded using a $f_{rep}$ of 35 kHz and $f_{dev}$ of 83.2 MHz after baseline correction. The rapid scan “wiggles” are clearly visible. A simulation of the transient magnetization using *blochsteady* is given for comparison.

*Saturation analysis*

It was necessary to determine the degree of saturation present when recording rapid scan spectra; therefore, a commercial Bruker X-band ElexSys E580 spectrometer equipped with a ELEXSYS Super High Sensitivity Probehead resonator (ER 4122 SHQ) was used to perform CW-EPR measurements of Ox071 in the presence and absence of oxygen (Fig S2*a*). The CW linewidth was measured over a wide range of B_1_ intensities for comparison to the linewidths observed via rapid scan using the EPRoC (Fig S2*b*). It was observed in both cases that the minimum linewidths obtained from CW measurements throughout the entire range of B_1_ intensities investigated was obtainable by frequency-swept rapid scan using the VCO of the EPRoC after Fourier deconvolution and Hilbert transformation.


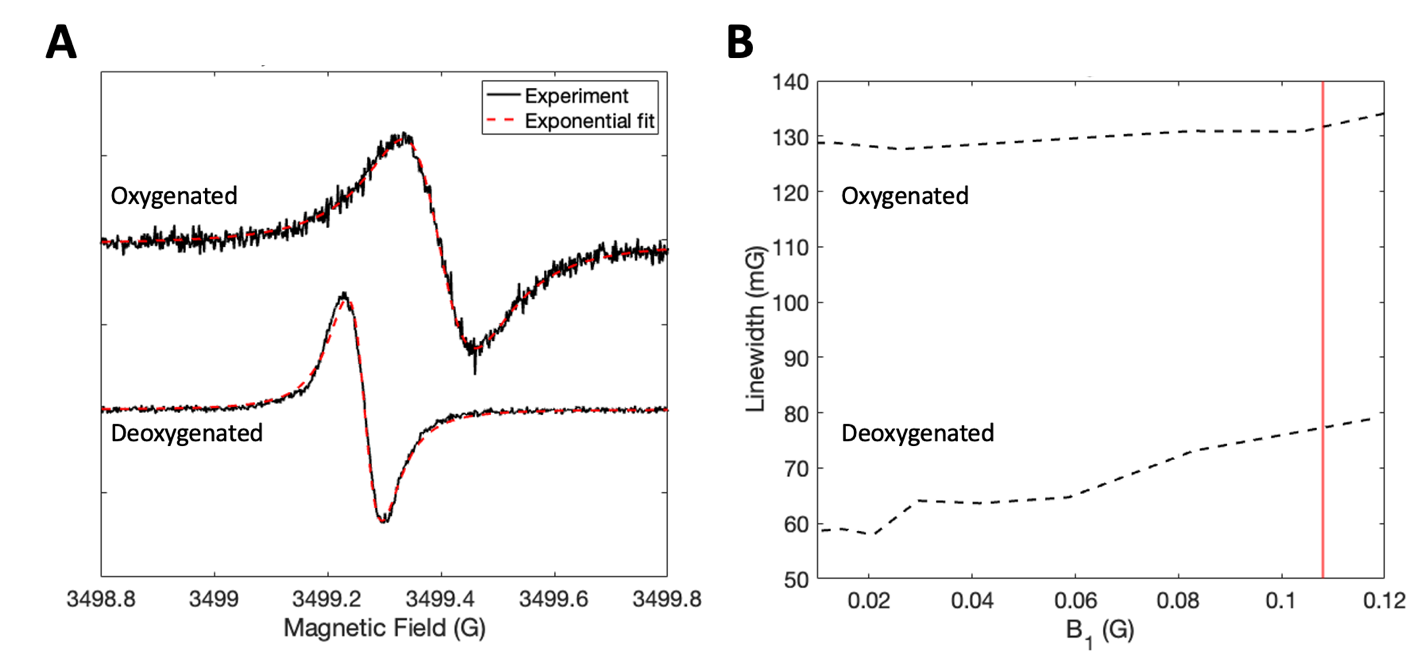


Figure S2: (a) CW-EPR spectra and associated fits for Ox071 solutions recorded using resonator-based conventional EPR in the presence and absence of oxygen. The resonance field varied between measurements due to slight variations in the microwave frequency after tuning the cavity. (b) The linewidths obtained in the presence and absence of oxygen as microwave power was increased after consideration of the conversion efficiency of the resonator. The B_1_ field intensity for each attenuation level was derived using a conversion factor of 2.4 G/W^1/2^. The anticipated intensity of the B_1_ generated by the VCO of the EPRoC is indicated by the vertical red line.

*SNR considerations*

There is an inherent trade-off between resonator-based field-swept rapid scan measurements and VCO-based frequency-swept rapid scan measurements. When considering resonator-based field-swept experiments, a primary determinant of SNR is the Q of the resonator. The resonator bandwidth is inversely related to Q such that for high quality resonators used in EPR (Q ≈ 10,000), the bandwidth is typically very low (< 5 MHz at X-band).^6^ This is additionally advantageous when operating at a fixed microwave frequency because the low bandwidth greatly suppresses any frequency components that lie outside of the resonator bandwidth (Fig S3*a*).^9^ When the field is swept to excite the full width of the spin packets present in the sample, the noise suppression of the resonator contributes to the improved SNR obtained. To acquire rapid scan spectra at fast sweep rates, the bandwidth of the resonator typically must be increased, thereby decreasing signal intensity by the lowering of Q while increasing the recorded noise over the increased bandwidth.

As has been shown in Ref^1^, the intrinsic SNR of the EPRoC detector in the frequency-sensitive detection mode is equivalent to, assuming only white frequency noise in the oscillator, the SNR of classical resonator-based EPR. Importantly, just like in a conventional resonator-based detector, both the signal and the noise display the same behavior vs. frequency, resulting in a constant SNR vs. frequency. However, in stark contrast to the resonator-based approach, the frequency noise is not shaped directly by the bandpass characteristic of the LC tank inside the oscillator but rather displays the behavior shown in Fig. S3b (adapted from Ref^10^) with a white frequency noise floor and a region that increases with a slope of 20 dB/dec. The white frequency noise originates from phase noise with a slope of –20 dB/dec, while the increasing frequency noise originates from the white phase noise floor. The corner frequency depends on the specific design parameters of the oscillator at hand. In the white frequency noise region, the spot SNR remains constant, and the time-domain SNR, i.e., signal over the integrated noise, decreases with the square root of the detection bandwidth. Importantly, the noise floor does not decrease, requiring only a constant-vs-frequency noise performance of the following LNA in this case. By contrast, the noise in conventional resonator-based EPR is shaped by the bandpass filter characteristic of the resonator, resulting in reduced signal and noise (but with a constant SNR) vs. offset frequency from the center frequency. The reduced noise makes it, in principle, harder to design a low-noise amplifier – assuming the LNA is directly connected to the resonator – whose added noise is below the intrinsic noise of the resonator since its noise would need to improve with increasing offset frequency for a constant spot noise figure. In conventional systems, the impedance is typically matched to $50 \Omega$, and this match degrades with offset frequency due to the finite bandwidth of the resonator and the matching network, resulting in similar noise figure issues for increased detection bandwidths, which are required for rapid-scan EPR.


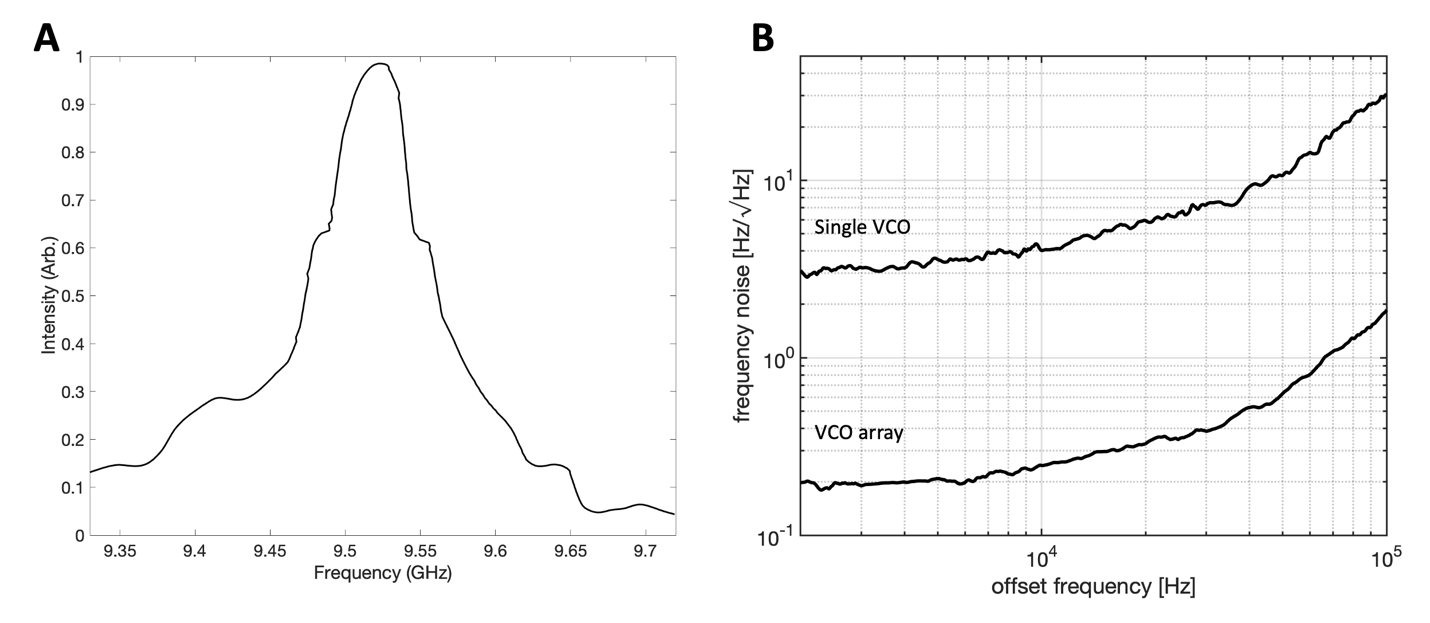

Figure S3: (a) The frequency response observed in a microwave resonator (Bruker MS-3), where all frequencies outside of the bandwidth are attenuated by the magnitude of the resonator matching. Data from Ref ^11^ digitized using WebPlotDigitizer.^12^ A 10-point 2^nd^ order Savitzky-Golay filter has been applied to the digitized data using *EasySpin*.^8^ (b) Frequency noise of a VCO-based single coil EPRoC (top) similar to the device used herein and an 8-coil VCO array EPRoC (bottom) as a function of offset frequency from the carrier. Adapted from Ref^10^.

References

(1) Anders, J. Fully-Integrated CMOS Probes for Magnetic Resonance Applications, EPFL, Lausanne, 2011. https://doi.org/10.5075/epfl-thesis-5154.

(2) Hassan, M. A.; Kern, M.; Chu, A.; Kalra, G.; Shabratova, E.; Tsarapkin, A.; MacKinnon, N.; Lips, K.; Teutloff, C.; Bittl, R.; Korvink, J. G.; Anders, J. Towards Single-Cell Pulsed EPR Using VCO-Based EPR-on-a-Chip Detectors. *Frequenz* **2022**, *76* (11–12), 699–717. https://doi.org/10.1515/freq-2022-0096.

(3) Khan, K.; Hassan, M. A.; Kern, M.; Lips, K.; Schwartz, I.; Plenio, M.; Jelezko, F.; Anders, J. A 12.2 to 14.9 GHz Injection-Locked VCO Array with an on-Chip 50 MHz BW Semi-Digital PLL for Transient Spin Manipulation and Detection. In *2022 IEEE 65th International Midwest Symposium on Circuits and Systems (MWSCAS)*; IEEE, 2022; Vol. 2022-August, pp 1–4. https://doi.org/10.1109/MWSCAS54063.2022.9859288.

(4) Künstner, S.; Chu, A.; Dinse, K.-P.; Schnegg, A.; McPeak, J. E.; Naydenov, B.; Anders, J.; Lips, K. Rapid-Scan Electron Paramagnetic Resonance Using an EPR-on-a-Chip Sensor. *Magnetic Resonance* **2021**, *2* (2), 673–687. https://doi.org/10.5194/mr-2-673-2021.

(5) Mitchell, D. G.; Quine, R. W.; Tseitlin, M.; Eaton, S. S.; Eaton, G. R. X-Band Rapid-Scan EPR of Nitroxyl Radicals. *Journal of Magnetic Resonance* **2012**, *214*, 221–226. https://doi.org/10.1016/j.jmr.2011.11.007.

(6) Eaton, G. R.; Eaton, S. S. Advances in Rapid Scan EPR Spectroscopy. In *Methods in Enzymology*; Academic Press Inc., 2022; Vol. 666, pp 1–24. https://doi.org/10.1016/bs.mie.2022.02.013.

(7) Eaton, G. R.; Eaton, S. S. Rapid-Scan Electron Paramagnetic Resonance. *eMagRes* **2016**, *5* (4), 1529–1542. https://doi.org/10.1002/9780470034590.emrstm1522.

(8) Stoll, S.; Schweiger, A. EasySpin, a Comprehensive Software Package for Spectral Simulation and Analysis in EPR. *Journal of Magnetic Resonance* **2006**, *178* (1), 42–55. https://doi.org/10.1016/j.jmr.2005.08.013.

(9) Lin, Y.-J.; Tseng, Y.-C.; Wu, T.-L. A Resonator-Based Suppressor for Mitigating the Noise Transfer on Metal Plates for Control of Electromagnetic Interference. *IEEE Microwave and Wireless Components Letters* **2016**, *26* (11), 906–908. https://doi.org/10.1109/LMWC.2016.2615002.

(10) Chu, A.; Schlecker, B.; Lips, K.; Ortmanns, M.; Anders, J. An 8-Channel 13GHz ESR-on-a-Chip Injection-Locked Vco-Array Achieving 200μM-Concentration Sensitivity. In *2018 IEEE International Solid - State Circuits Conference - (ISSCC)*; IEEE, 2018; pp 354–356. https://doi.org/10.1109/ISSCC.2018.8310330.

(11) Teucher, M.; Sidabras, J. W.; Schnegg, A. Milliwatt Three- and Four-Pulse Double Electron Electron Resonance for Protein Structure Determination. *Physical Chemistry Chemical Physics* **2022**, *24* (20), 12528–12540. https://doi.org/10.1039/D1CP05508A.

(12) Marin, F.; Rohatgi, A.; Charlot, S. WebPlotDigitizer, a Polyvalent and Free Software to Extract Spectra from Old Astronomical Publications: Application to Ultraviolet Spectropolarimetry. **2017**.
